# Long-term retinal imaging of a case of suspected congenital rubella infection

**DOI:** 10.1016/j.ajoc.2021.101241

**Published:** 2021-12-07

**Authors:** Christopher S. Langlo, Alana Trotter, Honey V. Reddi, Kala F. Schilter, Rebecca C. Tyler, Rupa Udani, Maureen Neitz, Joseph Carroll, Thomas B. Connor

**Affiliations:** a Department of Cell Biology, Neurobiology and Anatomy, Medical College of Wisconsin, Milwaukee, WI, USA; b Department of Ophthalmology and Visual Sciences, University of Wisconsin, Madison, WI, USA; c Precision Medicine Laboratory, Medical College of Wisconsin, Milwaukee, WI, USA; d Department of Ophthalmology, University of Washington, Seattle, WA, USA; e Department of Ophthalmology and Visual Sciences, Medical College of Wisconsin, Milwaukee, WI, USA

**Keywords:** Adaptive optics scanning light ophthalmoscopy, Rubella retinopathy, Pigmentary retinopathy, Cone photoreceptors

## Abstract

**Purpose:**

Many retinal disorders present with pigmentary retinopathy, most of which are progressive conditions. Here we present over nine years of follow up on a case of stable pigmentary retinopathy that is suspected to stem from a congenital rubella infection. Parafoveal cone photoreceptors were tracked through this period to gain insight into photoreceptor disruption in this pigmentary retinopathy.

**Methods:**

The patient was examined at 8 visits spanning a total of 111 months. Examination at baseline included clinical fundus examination, full-field electroretinography (ERG), kinetic visual field assessment (Goldmann), and best corrected visual acuity; all of these except ERG were repeated at follow up visits. Imaging was performed with fundus photography, spectral-domain optical coherence tomography (SD-OCT) and confocal adaptive optics scanning light ophthalmoscopy (AOSLO). For the latter four time points AOSLO imaging also included split-detector imaging.

**Results:**

There were no defects in hearing or cardiac health found in this patient. There were minimal visual deficits found at baseline, with mild rod suppression on ERG; best corrected visual acuity was 20/25 OD and 20/20 OS at baseline, which was stable throughout the follow-up period. Retinal thickness as measured by OCT was within the normal range, though foveal hypoplasia was present and outer nuclear layer thickness was slightly below the normal range at all time points. Cone density was relatively stable throughout the follow-up period. A number of cones were non-reflective when observed with confocal AOSLO imaging and density was markedly lower than expected values (foveal cone density was 43,782 cones/mm^2^ on average). Genetic analysis revealed no causative variations explaining the phenotype.

**Conclusions and Importance:**

This patient appears to have a stable pigmentary retinopathy. This case is likely due to a congenital insult, rather than progressive retinal disease. This finding of stability agrees with other reports of rubella pigmentary retinopathy. Imaging with AOSLO enabled observation of two notable phenotypic features. First is the observation of dark cones, which are seen in many retinal disorders including color vision defects and degenerative retinal disease. Second, the cone density is well below what is expected – this is especially interesting as this patient has near-normal visual acuity despite this greatly decreased number of normally-waveguiding cones in the fovea.

## 1 Introduction

Pigmentary retinopathies encompass a variety of retinal diseases. These conditions may be progressive, as in retinitis pigmentosa, or stationary as can be seen in congenital infection.^1^ The common feature in these diseases is the presence of darkly pigmented lesions observed in the fundus.^2^, ^3^, ^4^ The prognosis and expected management greatly differs between retinal degenerations and stationary defects, making proper diagnosis important for treatment planning and for the patient's expectations of the disease course.

One uncommon form of pigmentary retinopathy is caused by congenital infection with the rubella virus.^2^^,^^5^ While rare in the United States, rubella remains endemic in some regions of the world.^6^^,^^7^ This infection can cause a stationary retinopathy where pigmented retinal lesions are seen in addition to a classic triad of symptoms including hearing loss, heart defects, and cataract.^2^^,^^8^ This infection is difficult to detect, as the mother may experience no symptoms during the pregnancy yet have a latent infection that can be transferred to the child *in utero*.^9^ Additionally, due to strong immunization protocols in many countries, if the infection is not detected before vaccination, serum antibodies may not be an effective means of diagnosis. Finally, due to the live attenuated nature of the vaccine, non-vaccinated pregnant women cannot be vaccinated, and remain susceptible throughout their pregnancy. One recent report of four patients with congenital Rubella infection also showed pigmentary retinopathy.^10^ This and other reports have shown stationary retinal defects in patients who have known exposure to the rubella virus *in utero*.^2^^,^^8^ Here we present high resolution retinal imaging over a 111-month follow up period in a case of suspected rubella retinopathy.

## 2 Materials and methods

All study protocols were approved by the institutional review board at the Medical College of Wisconsin and adhered to the tenets of the Declaration of Helsinki. A 10-year-old boy born at term was noted to have pigmentary abnormalities in his fundus during routine eye screening. The examining optometrist diagnosed retinitis pigmentosa and referred the boy to a retina specialist who examined the boy and supported the diagnosis of retinitis pigmentosa. The boy's parents sought a second opinion at the retina clinic of Froedtert Memorial Lutheran Hospital Eye Institute (Milwaukee, WI) in February 2012. The fundus finding was confirmed during this visit, at which point he was considered for further detailed imaging. Written informed consent was obtained from the patient's parents as well as assent obtained from the patient prior to commencing the study. Written informed consent was obtained from the subject after his 18th birthday. The patient was followed from February 2012 through June 2021, with 8 total visits spanning 111 months, including clinical ophthalmic exam as well as high resolution retinal imaging.

### 2.1 Screening tests

Blood was drawn and genetic testing (John and Marcia Carver Laboratory, University of Iowa, Iowa City, IA) was initially performed to look for X-linked retinitis pigmentosa (*RPGR* and *RP2*) and autosomal recessive retinitis pigmentosa (*ABCA4, CERKL, CNGA1, CRB1, DHDDS, EYS, LRAT, MAK, NR2E2, PDE6B, RDH12, RLBP1, RPE65, SAG, TULP1, USH2A*) as well as testing of the *OPN1LW* and *OPN1MW* opsin genes (Maureen Neitz, University of Washington, Seattle WA). In response to a comment from a peer reviewer, whole exome sequencing was performed (Precision Medicine Laboratory, Medical College of Wisconsin, Milwaukee, WI) to look for pathogenic changes in almost 400 vision-associated genes (Supplemental Table 1). Syphilis blood testing was performed with an FTA-ABS test. Due to the patient having been vaccinated with the MMR vaccine and given his age, antibody titers were not assessed. Color vision was tested with the Neitz color vision test, the Colour Assessment and Diagnosis test and the Hardy-Rand-Rittler (HRR) test.

### 2.2 Clinical exam

Upon initial visit a physical exam including dilated fundus exam was performed on the patient after the following visual function tests were completed: visual acuity assessment (ETDRS), full-field electroretinography (ERG) and Goldmann kinetic visual fields. The acuity test, fundus exam and visual fields were then repeated at subsequent visits, while ERG was only performed at baseline. Static perimetry with a 30-2 Humphrey visual field test was acquired in the right eye (Carl Zeiss Meditec Inc., Dublin, CA USA) at the 68-month time point only.

### 2.3 Imaging

Fundus photography was captured using either a Visucam (Carl Zeiss Meditec Inc., Dublin, CA USA) or Optos wide field imager (Optos Inc., Dunfermline, Scotland, UK). Fundus autofluorescence was also obtained at baseline and at 30, 53, and 111 months. High resolution retinal imaging was performed at each visit. There was no phenotypic difference between eyes at baseline and for subsequent time points the right eye was chosen for further imaging and analysis. Images were acquired with Bioptigen (Leica Microsystems Inc., Buffalo Grove, IL USA) and Cirrus (Carl Zeiss Meditec Inc., Dublin, CA USA) optical coherence tomography (OCT) as well as adaptive optics scanning light ophthalmoscopy (AOSLO). OCT images included both volume and line scans, except for the 7-month time point when line scans were not acquired. Outer nuclear layer (ONL) thickness was measured at the fovea by creating a linear reflectivity profile and measuring the spacing between peaks corresponding to the external limiting membrane in the outer retina and the posterior boundary of the outer plexiform layer (given the presence of foveal hypoplasia). The AOSLO imaging was performed using a previously described custom device.^11^ Imaging from the first three dates included confocal imaging only, while the 2014 through 2021 dates included split-detection AOSLO as well.^12^

Cone density was measured using 80 × 80μm regions of interest (ROIs) with eccentricity measured relative to the foveal center as determined topographically by the Cirrus OCT. These regions were extracted at the foveal center and 0.65° in each the superior/nasal, superior/temporal, inferior/nasal and inferior/temporal directions. Follow-up images were scaled and manually aligned to the baseline image, and warped to the same manually aligned region from the reference image using bUnwarpJ^13^ to ensure the same cells were counted for each time point. Cone density was measured using the confocal images, with custom semi-automated cone detection software (Translational Imaging Innovations, Hickory, NC USA).^14^ The split-detector images were used to evaluate remnant cone structure in the dark regions seen on confocal images.

## 3 Results

### 3.1 Screening & clinical exams

**Fig. 1:**
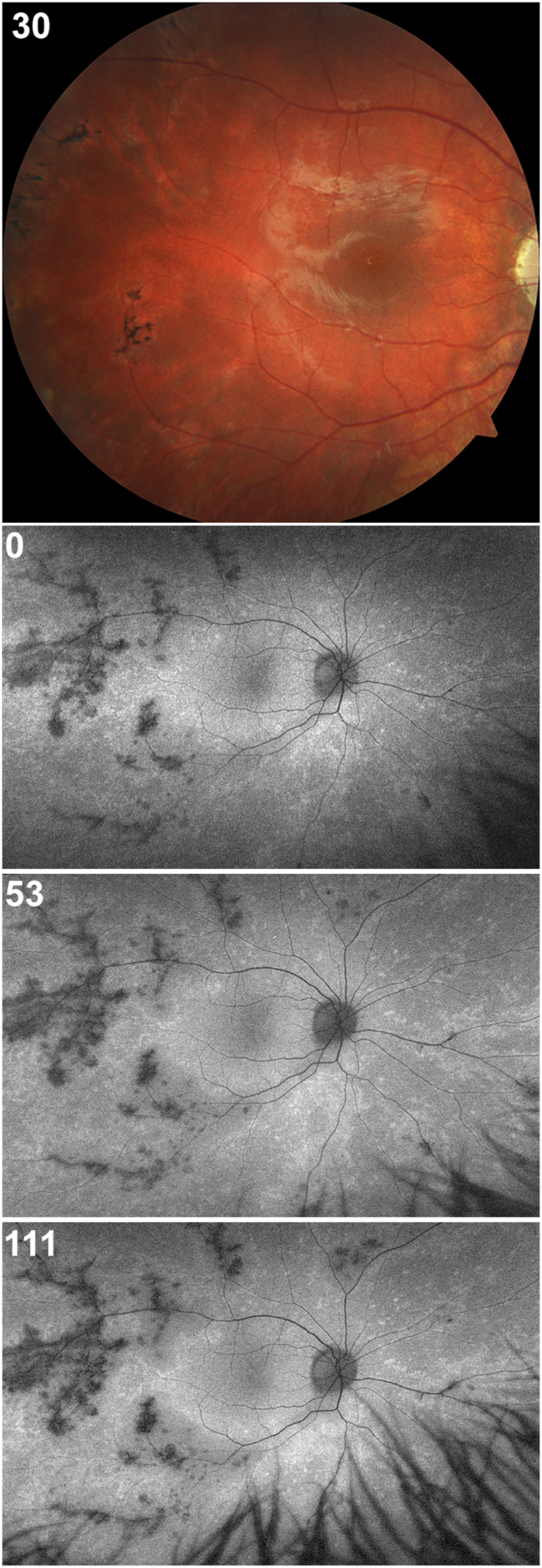
**Fundus appearance of the right eye**. Color fundus photo (*top panel*) shows pigmentary lesions in the temporal retina. Fundus autofluorescence (*bottom panels*) show hypoautofluorescent perivascular pigment deposits and punctate areas of hyperautofluorescence. Of note, there are minimal changes in the autofluorescence patterns over the 111-week follow-up. The time point for each image is indicated in the top left corner of each panel. (For interpretation of the references to color in this figure legend, the reader is referred to the Web version of this article.)

The patient reported no subjective visual disturbances, field defects or nyctalopia. There was no history of eye or head trauma nor known toxin exposure. No retinitis pigmentosa associated variations were discovered in any gene assayed, nor is there a family history of degenerative retinal disease. Whole exome sequencing revealed the presence of 3 heterozygous variants in genes associated with ocular pigmentation and 2 variants in genes associated with retinal degeneration. The genes/variants associated with ocular pigmentation included the hypomorphic allele in TYR, c.575C > A (p.S192Y), known to be associated with foveal hypoplasia and the two variants of unknown clinical significance in HERC2, c.14015C > T (p.T4672 M) and SLC38A8, c.954-4C>T splice site variant. These findings are consistent with the patient's overall low level of pigmentation. The 2 variants identified in genes associated with retinal degeneration include a pathogenic nonsense variant in the MMACHC gene, c.331C > T (p.R111*), associated with recessive metabolic disease and a variant of unknown clinical significance in KIF11, c.2923-7_2923-5delCTT, which is associated with familial exudative vitreoretinopathy and has also been reported to be associated with pigmentary retinopathy. See Supplemental Table 1 for a summary of genetic results and genes surveyed. Additionally, congenital syphilis infection was ruled out via nonreactive FTA-ABS blood test. Laboratory testing also revealed a normal complete blood count and normal levels of plasma ornithine and phytanic acid. No color vision abnormalities were detected. Moreover, the X-linked opsin gene array was normal, comprised of a single *OPN1LW* gene (exon 3 = LIAIS) followed by two *OPN1MW* genes (exon 3 = MVVVA). While certain exon 3 haplotypes have been associated with altered cone reflectivity,^15^, ^16^, ^17^ neither sequence in this patient is known or would be expected to cause altered cone reflectivity.^18^^,^^19^ This patient showed no symptoms of hearing loss, heart defect, or anterior segment ophthalmic defects. Best corrected visual acuity was found to be 20/25 OD and 20/20 OS throughout the follow up period. Fundus examination revealed pigmentary deposits distributed as patches extending from beyond the equator anteriorly to the edge of the arcades temporally and beyond the optic disc nasally and showed some mild optic disc atrophy but was otherwise unremarkable (Fig. 1). Fundus autofluorescence showed hypoautofluorescence in areas of perivascular pigmentary deposits. There were also areas of punctate hyperautofluorescence scattered in a mostly perivascular pattern. These findings remained stable throughout the follow up period (Fig. 1). Electroretinography showed a normal cone response with 30 Hz flicker, but a diffusely depressed though not extinguished rod response, more prominent in the right eye.

### 3.2 Visual fields

Kinetic fields showed no defects at baseline. There was a scotoma observed at the third visit in the superior-temporal region of the right eye, though this was not seen at any other time point. Beginning at the 40-month time point the sensitivity in the temporal retina of the right eye was slightly decreased, and remained so through the 68 month time point. Static fields acquired at 68 months showed a decreased sensitivity in the temporal retina as well. No visual fields were acquired at the 111-month time point.

### 3.3 OCT imaging

**Fig. 2:**
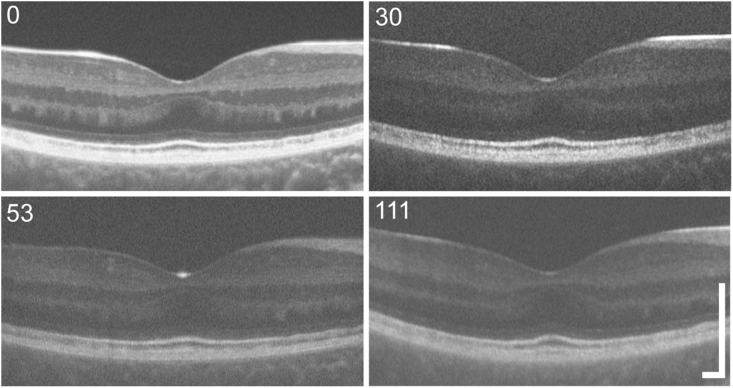
**Stable optical coherence tomography phenotype**. Shown here are four OCT line scans acquired over the 111-week follow-up. Notably there is foveal hypoplasia present in all images, with the ganglion cell layer, inner plexiform layer, inner nuclear layer, and outer plexiform layer persistent through the fovea. The outer retinal bands do not show any significant disruption at the fovea, though there is subtle mottling of the photoreceptor bands in the nasal retina. The overall appearance of the retina was similar across all images. The time point for each image is indicated in the top left corner of each image. Scale bars – 250 μm.

OCT imaging revealed foveal hypoplasia in both eyes with the ganglion cell, inner plexiform, inner nuclear, and outer plexiform layers being persistent through the fovea (Fig. 2). The right eye was chosen for quantitative analysis. At baseline central total retinal thickness was found to be 210 μm which is within the range for non-diseased retinas.^20^^,^^21^ Outer nuclear layer thickness at baseline was 85 μm, which is slightly below the normal range.^22^^,^^23^ These thickness values were slightly higher at 18 months (217 μm and 92 μm respectively) and then remained unchanged after at the two most recent visits (221 μm and 93 μm respectively at 68 months and 229 μm and 91 μm, respectively at 111 months). There were no gross changes in perifoveal structure observed on OCT throughout the follow up period (Fig. 2). There is a region of outer retinal atrophy with loss of photoreceptor layers in the temporal retina that extends superiorly and inferiorly from the nasal-temporal meridian. This region appeared stable through the follow up period.

### 3.4 AOSLO imaging

**Fig. 3:**
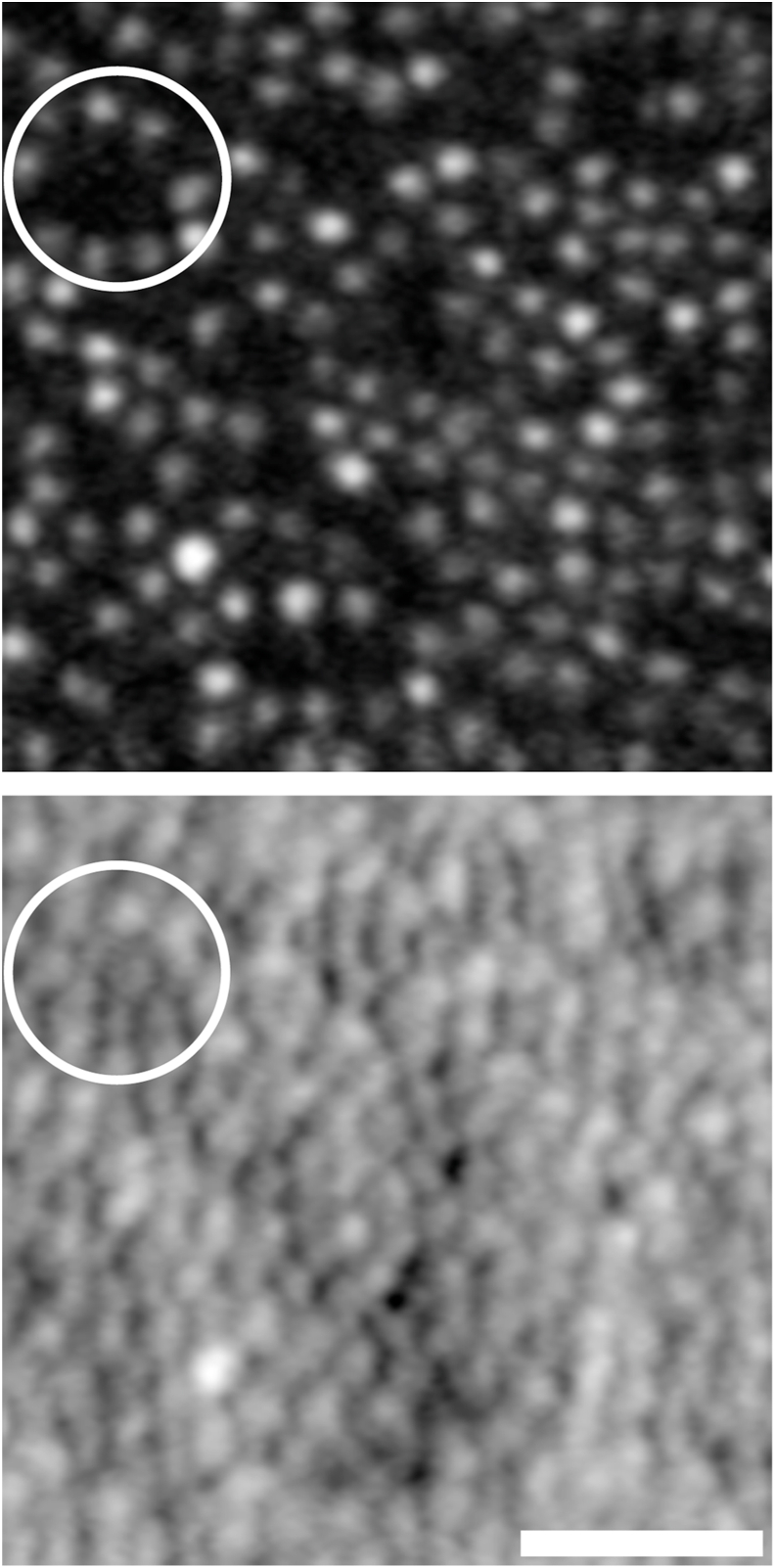
**Adaptive optics shows dark and bright cones**. Confocal (*top*) and split-detector (*bottom*) images of the same retinal location at the 40-month time point. The circled region highlights an area with dark cones in the confocal image, while there are remnant cone inner segments visible in the split-detector image. Scale bar – 25 μm.

Imaging with AOSLO revealed a non-contiguous mosaic of cones with dark gaps among them (Fig. 3). These dark gaps were found to contain cone photoreceptor inner segments with split-detector AOSLO (Fig. 3), indicating the presence of cone cells with altered reflectivity presumably due to disrupted structure. Cone density values (assessed on confocal AOSLO images) were lower than those found in normal retinas across all locations (Table 1).

**Table 1:** Density values (Cones/mm^2^) for each time point, bottom row is the mean normal value in these locations. Locations are about 0.65° away from the foveal center. ST: Superior/temporal, SN: Superior/nasal, IT: Inferior/temporal, IN: Inferior/nasal.

Months	ST	SN	IT	IN	Center
0	27492	41877	25414	27651	42516
7	28451	45074	26852	27332	45873
18	29090	40438	26053	27172	43795
30	28451	43155	28131	31807	41717
40	29244	41877	24934	23496	41078
53	29090	46224	24615	28291	46512
68	32127	45553	27012	28610	44274
111	28458	40879	25785	27986	44495
Normal^24^	73914	75418	70204	70575	166854^43^

**Fig. 4:**
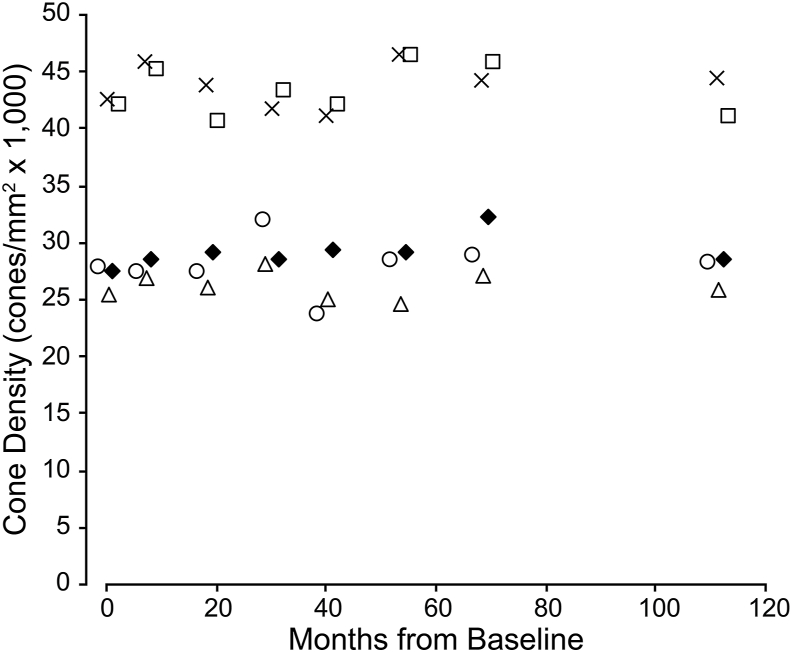
**Longitudinal cone densities**. Shown are the density values for each of the five ROIs during the follow up period (measured using confocal AOSLO images). There is no significant change in density observed during the follow up period. Data points were displaced horizontally for visibility. Diamond: superior/temporal, Square: superior/nasal, Triangle: inferior/temporal, X: inferior/nasal, Circle: center.

**Fig. 5:**
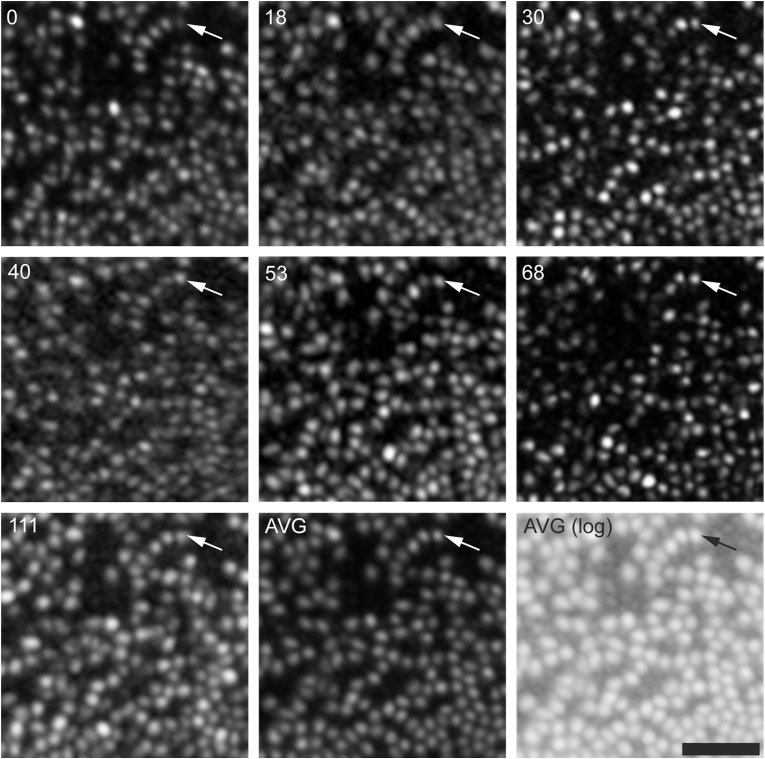
**Cone images are stable over follow up period.** A series of confocal images in the superior-temporal direction roughly 0.65° from the fovea. The pattern of dark gaps between reflective cones appears stable across all time points. Similarly, the same reflective cells can be identified across all images. The AVG image (*bottom middle panel*) is an unweighted average of all time points, with a log-scaled version of this image shown in the bottom right panel. Arrow indicates location of a row of cells that are easily identified across all images. The time point for each image is indicated in the top left corner. Scale bar – 25 μm.

Follow up imaging with AOSLO of the right eye showed cone density to be stable at the foveal center, and at locations about 0.65° in the superior-temporal, superior-nasal, inferior-temporal and interior-nasal directions (Fig. 4). Changes in density were within known measurement error (Fig. 4).^24^ Individual cones can be tracked across all time points at each ROI, as is shown in Fig. 5. Densities measured at each ROI with split-detector images were on average 24% greater than the confocal cone densities, as expected due to the non-reflective cones present in these ROIs.

## 4 Discussion

No ophthalmic problems aside from the pigmentary retinopathy were discovered in this patient. There was no history of physical or chemical insult that may have led to this appearance. He does have a minor form of Von Willebrand disease which manifested as a mild petechial rash and easy bruising as an infant, though this would not be expected to cause the pigmentary retinopathy. The retinopathy was found in isolation from visual symptoms, with his suppressed rod ERG being the only clinically detected retinal deficiency. No definitive evidence was found of infectious disease.

Whole exome sequencing was performed as initial genetic testing was limited. Notably there are three variations in pigmentation related genes (*TYR, HERC2, SLC38A8*). As *TYR*^25^ and *SLC38A8*^26^ have been previously associated with foveal hypoplasia, this may explain the foveal hypoplasia seen in our patient. Furthermore there is a pathogenic variant in the *MMACHC* gene which is associated with methylmalonic aciduria and homocystinuria. This condition has been associated with retinopathy and rod-cone dystrophy,^27^ however it is a recessive condition with other systemic manifestations – the single allele affected in this patient as well as a lack of systemic illness suggest this change is unrelated to the retinopathy reported here. Most interestingly there is a variation of unknown significance in *KIF11,* defects in which can cause autosomal dominant disease. While defects in this gene have been associated with familial exudative vitreoretinopathy,^28^ this condition is inconsistent with our patient's presentation. Variations in *KIF11* have also been linked to some cases of chorioretinal dystrophy.^29^^,^^30^ These patients have been reported to have a primarily central macular dystrophy which is not seen in the patient reported here.^30^ Given KIF11 is a ciliary protein, it is possible that the change underlies the variably altered cone waveguiding/reflectance (but not the gross pigmentary phenotype). Similar reflectivity changes are seen in patients with Usher syndrome.^31^

These results are slightly puzzling, as a stationary pigmentary retinopathy with no obvious visual symptoms and no defects in other body systems is uncommon. Indeed, the variations of interest reported here are all most often associated with systemic effects not seen in this patient with the exception of decreased overall pigmentation. There are few causes of congenital pigmentary retinopathy, and inherited retinal degeneration is unlikely given the lack of evidence of progression and the phenotypic inconsistency with the genetic defects discovered. Congenital infection is a known cause of this type of retinal phenotype, though in this case definitive diagnosis was challenging given the presentation was far removed from the perinatal period. We believe that the pigmentation and the subsequently discovered disruption of cone structure seen in this case are consistent with a subclinical rubella infection that occurred prior to or shortly after birth in this patient, with an immune response bolstered by the patient's mother fighting off infection before more damage was done – although a definitive diagnosis is not possible.

There is no evidence of any progression observed with imaging. Though there was a scotoma present at one time point in the kinetic field, its previous absence, and disappearance in subsequent testing, paired with the patient's young age at the time and the subjective nature of the test, point toward this scotoma as a likely artifact of a lapse in the patient's attention to the task. The decreased sensitivity at later time points may be indicative of true visual change, or they may have been stable throughout but, as with the scotomas, may be an indication of increased attentional capacity of the patient. OCT imaging was stable across the entire follow up period, showing no large-scale retinal remodeling over 111 months. The region of apparent outer retinal degeneration in the temporal, superotemporal and inferotemporal retina corresponds to the defects seen on visual fields. This area appears stable though and has likely been present since birth.

The stability of the cone population across all ROIs suggests no cellular level changes are occurring near the fovea. The cones observed across more than nine years of follow up can be tracked across all images, and do not change. Cone reflectivity has been shown to change over time in normal retinas,^32^^,^^33^ leading to the question of whether the dark cones are always non-reflective. While the cones observed here do fluctuate in intensity as in a normal retina, the non-reflective cones were persistently non-reflective throughout the follow-up period. However, the split-detector images confirm the presence of remnant cone inner segment structures in these dark gaps in the mosaic.

The findings in this case have important implications for understanding other retinal disease. In many conditions it has been presumed that these dark cones are non-functional,^31^^,^^34^^,^^35^ and AO-based microperimetry studies support this in an individual with red-green color blindness.^36^ However in other cases, retinal locations lacking normally-reflective cone structure have been shown to be sensitive to light stimulus.^37^^,^^38^ Our patient has normal or near normal acuity, though the density of the cones at the fovea is well below the normal range. This disconnect suggests that the non-reflective cones may be contributing to this patient's vision. However, the density of all cones, including the non-reflective cones seen on split-detector, is still below the normal range. The hypoplasia seen in this patient is likely related to the variants in pigmentary genes. Foveal hypoplasia has been linked to decreased foveal cone packing in other conditions including albinism and aniridia – though visual acuity is generally decreased to a greater degree in these cases.^39^^,^^40^ This individual with little to no visual deficit but a reduced foveal cone population supports the hypothesis that the number of cones in the normal retina greatly exceeds the number needed for normal acuity. It has been reported that patients with retinal degeneration can lose up to 50% of the foveal cones before visual acuity deficits are noted.^41^ A study of an individual with a congenital red-green color vision defect revealed a mottled foveal cone mosaic with reduced density but no visual acuity deficit.^36^^,^^42^ In our case, there is a greater than 50% reduction in cone density at the fovea with BCVA of 20/25. In light of this result, it may be that therapies to restore cone function in other conditions may have the potential to result in near-normal visual capability even with significantly reduced cone populations.

## 5 Conclusions

This examination of cone photoreceptors in presumed rubella retinopathy shows that despite a developmental disruption, parafoveal cone structure is stable throughout a period of over nine years. This stability over a long period of time contributes to the existing literature that shows rubella retinopathy is a stationary condition. Examination of these cells revealed a reduced cone density, with numerous non-reflective cones whose presence was confirmed with split-detector imaging. Despite these retinal changes the normal vision in this subject suggests either that a greatly reduced cone population can still provide normal visual function, or many of these dark cones are functional.

## Consent

The subject and/or the subject's guardians provided written informed consent to all study procedures and the publishing of these data.

## Funding

This research was supported by the 10.13039/100000002National Institutes of Health 10.13039/100000053National Eye Institute under the awards R01EY017607, P30EY001931, P30EY001730, T32EY014537, by the 10.13039/100000057National Institute of General Medical Sciences of the NIH under award number T32GM080202, and by the 10.13039/100006108National Center for Advancing Translational Sciences of the NIH under award number UL1TR001436. This investigation was conducted in part in a facility constructed with support from a Research Facilities Improvement Program, grant number C06RR016511 from the 10.13039/100000097National Center for Research Resources, NIH. The content is solely the responsibility of the authors and does not necessarily represent the official views of the National Institutes of Health. This research was also supported by a 10.13039/100001818Research to Prevent Blindness unrestricted departmental grant to the 10.13039/100007812University of Washington Department of Ophthalmology.

## Authorship

All authors attest that they meet the current ICMJE criteria for Authorship.

## CRediT authorship contribution statement

**Christopher S. Langlo:** Conceptualization, Methodology, Formal analysis, Investigation, Writing – original draft, Writing – review & editing. **Alana Trotter:** Investigation, Writing – review & editing. **Honey V. Reddi:** Formal analysis. **Kala F. Schilter:** Formal analysis. **Rebecca C. Tyler:** Formal analysis. **Rupa Udani:** Formal analysis. **Maureen Neitz:** Formal analysis, Resources. **Joseph Carroll:** Conceptualization, Methodology, Writing – review & editing, Supervision, Project administration, Funding acquisition. **Thomas B. Connor:** Conceptualization, Methodology, Writing – review & editing, Supervision, Project administration, Funding acquisition.

## Declaration of competing interest

J. Carroll receives research support from OptoVue, AGTC, and Meira GTx, has financial interest in Translational Imaging Innovations, and is a paid consultant for Meira GTx. The following authors have no financial disclosures: CSL, AT, HR, KS, RT, RU, MN, TBC.
