# Supplementary material for: Long-term retinal imaging of a case of suspected congenital rubella infection
Source: Am J Ophthalmol Case Rep. 2021 Dec 7;25:101241. doi: 10.1016/j.ajoc.2021.101241 (PMC8688893; doi:10.1016/j.ajoc.2021.101241)
Supplement: Multimedia component 1 [file mmc1.docx]

**Supplemental Table 1**. Summary of Genetic Findings

| **Gene** | **Variation** | **Pathogenicity** |
| --- | --- | --- |
| *HERC2* | c.14015C>T; p.T4672M | Variation of unknown significance |
| *KIF11* | c.2923-7 2923-5delCTT | Variation of unknown significance |
| *MMACHC* | c.331C>T; p.R111* | Pathogenic |
| *SLC38A8* | c.954-4C>T | Variation of unknown significance |
| *TYR* | c.575C>A; p.S192Y | Hypomorphic allele |

Genes targeted for analysis: *ABCA4, ABCC6, ABHD12, ACO2, ADAM9, ADAMTS18, ADAMTS20, ADGRV1, ADIPOR1, AGBL5, AHI1, AHR, AIPL1, ALMS1, AP3B1, AP3D1, ARCN1, ARHGAP25, ARHGAP35, ARHGEF18, ARL13B, ARL2BP, ARL3, ARL6, ARMC9, ARSG, ASIP, ATF6, ATOH7, ATP13A2, ATP7A, ATRN, B9D1, B9D2, BBIP1, BBS1, BBS10, BBS12, BBS2, BBS4, BBS5, BBS7, BBS9, BEST1, BLOC1S1, BLOC1S3, BLOC1S4, BLOC1S6, BMPR1B, C1QTNF5, CFAP410, PCARE, CPLANE1, C8orf37, C10orf11, CA4, CABP4, CACNA1A. CACNA1F, CACNA2D4, CAPN5, CASK, CC2D2A, CD63, CDH23, CDH3, CDHR1, CDKN2A, CEP104, CEP120, CEP164, CEP19, CEP250, CEP290, CEP41, CEP78, CERKL, CHM, CIB2, CISD2, CLN3, CLRN1, CNGA1, CNGA3, CNGB1, CNGB3, CNNM4, COL11A1, COL11A2, COL18A1, COL2A1, COL9A1, COL9A2, COL9A3, CPE, CRB1, CRX, CSPP1, CTC1, CTNNA1, CTNNB1, CWC27, CYP26A1, CYP4V2, DHDDS, DHX38, DRAM2, DRD2, DTHD1, DTNBP1, EDN3, EDNRB, EFEMP1, EGFR, ELOVL4, EMC1, ESPN, EYS, FAM161A, FDXR, FLVCR1, FRMD7, FZD4, GAS1, GNA11, GNA13, GNAT1, GNAT2, GNAQ, GNB3, GNPAT, GNPTG, GPR143, GPR179, GRK1, GRM6, GUCA1A, GUCY2D, HARS, HERC2, HGD, HGSNAT, HK1, HMX1, HPS1, HPS3, HPS4, HPS5, HPS6, IDH3A, IDH3B, IFT140, IFT172, IFT27, IFT81, IHH, IMPDH1, IMPG1, IMPG2, INPP5E, INVS, IQCB1, IRF4, JAG1, KCNJ13, KCNV2, KIAA0556, KIAA0586, KIAA0753, KIAA1549, KIF11, KIF7, KIT, KITLG, KIZ, KLHL7, KRT1, KRT17, KRT2, LCA5, LRAT, LRIT3, LRP2, LRP5, LYST, LZTFL1, MAK, MANBA, MC1R, MED1, MITF, MERTK, MFN2, MFRP, MFSD8, MKKS, MKS1, MLANA, MLPH, MMACHC, MREG, MTTP, MVK, MYO5A, MYO7A, NDP, NEK2, NMNAT1, NPHP1, NPHP3, NPHP4, NR2E3, NR2F1, NRL, NYX, OAT, OCA2, OFD1, OPA1, OPA3, OTX2, P3H2, PAH, PANK2, PAX2, PAX3, PAX6, PCDH15, PCYT1A, PDE6A, PDE6B, PDE6C, PDE6D, PDE6G, PDE6H, PDGFC, PDZD7, PEX1, PEX10, PEX11B, PEX12, PEX13, PEX14, PEX16, PEX19, PEX2, PEX26, PEX3, PEX5, PEX6, PEX7, PHYH, PISD, PITPNM3, PLA2G5, PMEL, PNPLA6, POC1B, POMC, POMGNT1, PRCD, PRDM13, PROM1, PRPF3, PRPF31, PRPF4, PRPF6, PRPF8, PRPH2, PRPS1, PTPN22, RAB27A, RAB28, RAB3A, RAB32, RAB38, RAB7A, RAX2, RBP1, RBP3, RBP4, RCBTB1, RD3, RDH11, RDH12, RDH5, REEP6, RGR, RGS9, RGS9BP, RHO, RIMS1, RLBP1, ROM1, RP1, RP1L1, RP2, RPE65, RPGR, RPGRIP1, RPGRIP1L, RS1, RTN4IP1, SACS, SAG, SAMD11, SCAPER, SCLT1, SDCCAG8, SEMA4A, SERPINF1, SETX, SHROOM2, SLC24A1, SLC24A4, SLC24A5, SLC25A46, SLC38A8, SLC45A2, SLC7A14, SNRNP200, SOX10, SPATA7, SPP2, SRD5A3, TCTN1, TCTN2, TCTN3, TEAD1, TIMM8A, TIMP3, TMEM107, TMEM126A, TMEM138, TMEM216, TMEM231, TMEM237, TMEM67, TOPORS, TPCN2, TRAF3IP1, TREX1, TRIM32, TRPM1, TSPAN12, TTC21B, TTC8, TTLL5, TTPA, TUB, TUBB4B, TULP1, TYR, TYROBP, TYRP1, UNC119, USH1C, USH1G, USH2A, VCAN, VLDLR, VPS13B, WDPCP, WDR19, WFS1, WHRN, YME1L1, ZNF408, ZNF423, ZNF513*
